# Effectiveness of four deconstructive meditative practices on well-being and self-deconstruction: study protocol for an exploratory randomized controlled trial

**DOI:** 10.1186/s13063-023-07151-0

**Published:** 2023-02-20

**Authors:** Javier García-Campayo, Rinchen Hijar-Aguinaga, Yolanda López-Del-Hoyo, Rosa Magallón-Botaya, Selene Fernández-Martínez, Alberto Barceló-Soler, Joaquim Soler-Ribaudi, Jesus Montero-Marin

**Affiliations:** 1 Aragon Institute for Health Research, IIS Aragon, Saragossa, Spain; 2 Department of Medicine, Psychiatry and Dermatology, Faculty of Medicine, University of Zaragoza, Saragossa, Spain; 3 Department of Psychology and Sociology, Faculty of Social and Human Sciences, University of Zaragoza, Saragossa, Spain; 4 Primary Care Prevention and Health Promotion Research Network (RedIAPP), Saragossa, Spain; 5 University of Zaragoza, Saragossa, Spain; 6 Arrabal Primary Care Health Center, Aragon Health Service, Saragossa, Spain; 7 Navarra Medical Research Institute (IdiSNA), Pamplona, Spain; 8 Department of Psychiatry, Hospital de La Santa Creu I Sant Pau, Barcelona, Spain; 9 Universitat Autònoma de Barcelona (UAB), Barcelona, Spain; 10 Centro de Investigación Biomédica en Red de Salud Mental, Institut d’Investigació Biomèdica-Sant Pau (IIB-SANT PAU), CIBERSAM, Barcelona, Spain; 11 Department of Psychiatry, Warneford Hospital, University of Oxford, Oxford, OX37JX UK; 12 Teaching, Reseach and Innovation Unit, Parc Sanitari Sant Joan de Déu, Sant Boi de Llobregat, 08830 Spain; 13 Consortium for Biomedical Research in Epidemiology & Public Health, CIBER Epidemiology and Public Health - CIBERESP, Madrid, 28029 Spain

**Keywords:** Deconstructive meditation practices, Mindfulness, Randomized controlled trial, Wellbeing, Self-deconstruction, General population

## Abstract

**Introduction:**

The efficacy of interventions based on mindfulness and compassion has been demonstrated in both clinical and general population, and in different social contexts. These interventions include so-called attentional and constructive meditation practices, respectively. However, there is a third group, known as deconstructive meditation practices, which has not been scientifically studied. Deconstructive practices aim to undo maladaptive cognitive patterns and generate knowledge about internal models of oneself, others and the world. Although there are theoretical and philosophical studies on the origin of addiction to the self or on the mechanisms of action associated with the deconstruction of the self, there are no randomized controlled trials evaluating these techniques in either a healthy population or clinical samples. This study aims to evaluate the effect of three deconstructive techniques by comparing them to mindfulness in the general population.

**Methods and analysis:**

A randomized controlled clinical trial will be conducted with about 240 participants allocated to four groups: (a) mindful breathing, (b) prostrations, according to Tibetan Buddhist tradition; (c) the Koan Mu, according to Zen Buddhist tradition; and (d) the mirror exercise, according to Toltec tradition. The primary outcome will be the qualities of the non-dual experience and spiritual awakening, measured by the Nondual Embodiment Thematic Inventory, assessed at pre- and post-treatment and at 3- and 6-month follow-ups. Other outcomes will be mindfulness, happiness, compassion, affectivity and altered state of consciousness. Quantitative data will be compared using mixed-effects linear regression models, and qualitative data will be analysed through thematic analysis and using the constant comparative method from grounded theory.

**Ethics and dissemination:**

Approval was obtained from the Research Ethics Committee of Aragon, Spain. The results will be submitted to peer-reviewed specialized journals, and brief reports will be sent to participants on request.

**Trial registration:**

ClinicalTrials.gov NCT05317754. Registered on August 2,2022.

## Background

According to Kabat-Zinn [1], “mindfulness is the practice of purposely bringing one’s attention to experiences occurring in the present moment without judgment”, a skill that can be developed through attentional meditation. Since he started to teach mindfulness in 1979 at a hospital associated with the University of Massachusetts, mindfulness has demonstrated efficacy for increasing psychological and physical well-being in healthy adults in general [2], and in specific environments such as clinical settings [3], the workplace [4] and schools [5, 6].

In the early years of the twenty-first century, another kind of meditative technique, known as compassion, emerged in the West. Goetz et al. [7] defined compassion as the feeling that arises when witnessing another’s suffering and motivates a subsequent desire to help. Systematic reviews and meta-analyses indicate that compassion has an important role to play in the treatment of disorders such as depression and anxiety and the improvement of well-being in healthy individuals [8–10].

The large number of meditation types and the great differences that set them apart led authors such as Dahl et al. [11] to develop a now widely used classification, based on cognitive neuroscience and clinical psychology, which comprises three groups of meditation types:

- Attentional meditations. These types train a variety of processes related to the regulation of attention, with mindfulness being the best-known type in this group. The authors propose that a shared characteristic of all these meditation practices is the systematic training of the capacity to intentionally initiate, direct and/or sustain these attentional processes while strengthening the ability to be aware of the processes of thinking, feeling and perceiving.
- Constructive meditation. These types include a variety of meditation practices that strengthen psychological patterns that foster well-being. In contrast to attentional practices, which often focus on simply monitoring cognitive and affective patterns, constructive meditation involves systematically altering the content of thoughts and emotions. Compassion would be the most widely studied technique within this group.
- Deconstructive meditation. These types aim to undo maladaptive cognitive patterns by exploring the dynamics of perception, emotion and cognition, and by generating insights into one’s internal models of the self, others and the world. The authors suggest that a central mechanism in this kind of meditation is self-inquiry, which is the process of investigating the dynamics and nature of conscious experience.

In contrast with the great amount of scientific research on attentional and constructive meditations, deconstructive meditation types have received little attention from neuroscientists [12]. This is surprising, given that deconstruction of the self forms the core of meditative practices in a number of major Eastern contemplative traditions, such as Buddhism, Vedanta Advaita and Taoism [13]. In fact, there is a trend away from the stringent frame of mindfulness and meditation to a wider concept of contemplative sciences [14], in which deconstruction of the ego would be one of the most important goals.

Moreover, evolutionary social psychology has also shown concern for the fact that human beings have an evolved capacity for self-awareness, along with a propensity to focus primarily on their own welfare. Although this focus has clear adaptive functions, such as physical preservation and self-regulation, this pervasive egoic mindset, in an increasingly crowded and interdependent world, is associated with high personal and societal costs. This is the basis of rising interest in hypo-egoic phenomena in psychology [15].

Despite the publication of a number of theoretical and philosophical papers on the origin of ego addiction [16] and the mechanisms of action associated with deconstruction of the ego [17, 18], no randomized controlled trials have been conducted to assess these techniques either in a healthy population or in clinical samples. This is the first exploratory randomized controlled trial to evaluate the effect of several different deconstructive meditation techniques on healthy individuals. Our hypothesis is that all four interventions would be effective for decreasing the ego perspective in the general population, and more specifically, that the three specific deconstructive interventions would be more effective than the control attentional mindfulness technique.

## Methods

### Study design

Participants will be randomly allocated to a four-armed randomized controlled clinical trial with four conditions: (a) standard mindfulness alone, using the mindful breathing technique mindfulness of breathing, which will be considered the control condition; (b) prostrations, according to Tibetan Buddhist tradition; (c) the Koan Mu, according to Zen Buddhist tradition; and (d) the mirror exercise, according to Toltec tradition. They will be assessed at pre- and post-treatment and 3- and 6-month follow-ups. This study will conform to the Consolidated Standards of Reporting Trials (CONSORT) statement (http://www.consort-statement.org) [19] for reporting trials and the Standard Protocol Items: Recommendations for Intervention Trials (SPIRIT) guidelines [20] for clinical trial protocols. The study’s trial registration number is ClinicalTrials.gov NCT05317754.

### Study population, recruitment and eligibility criteria

This randomized controlled clinical trial will be conducted within the Master’s in Mindfulness programme and supervised by the Chair of Contemplative Sciences of the University of Zaragoza. Participants will be healthy adults recruited online from the general population of Spain and Spanish-speaking countries.

The participants will be recruited through several different social media (e.g. Twitter, Facebook and LinkedIn) and university newsletters (specifically, as part of the university’s Master’s in Mindfulness programme). When participants contact the research team, the study will be explained by one of the researchers, and if they agree to participate, an informed consent form containing the main aim of the study, the name of the study leader and contact details will be provided. In order to prevent a high dropout rate, the research team will emphasize that once the study is completed, participants will be able to access the other study interventions, as well as being able to request a report with the results of the study.

**Fig. 1:**
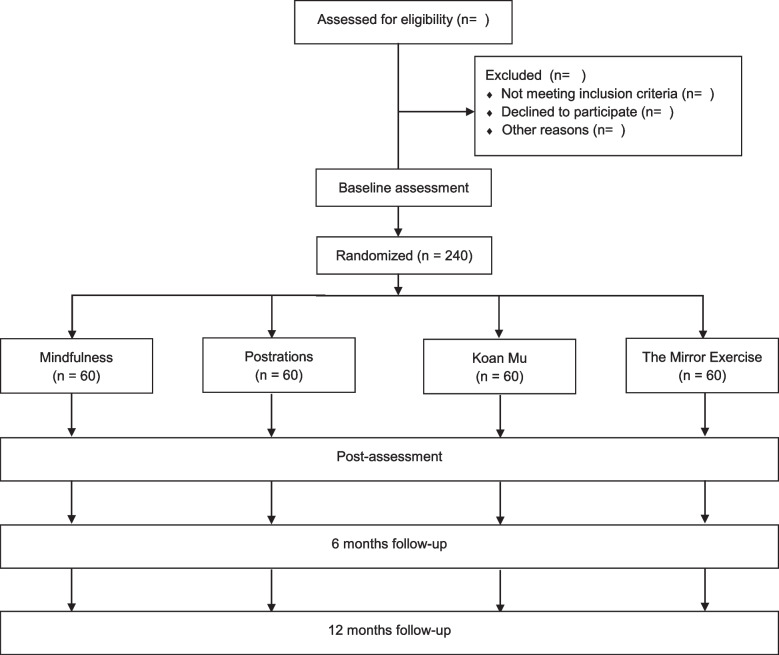
Study flowchart

**Fig. 2:**
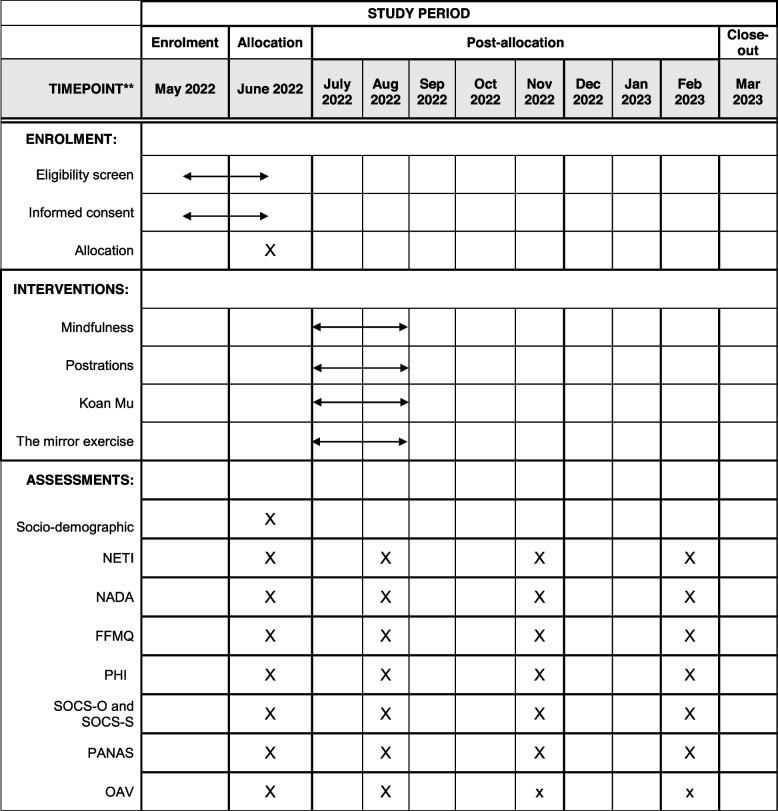
Schedule of enrolment, interventions and assessments

Participants will subsequently undergo a clinical interview and fill out the paper-and-pencil screening survey describing their sociodemographic and clinical characteristics. This first data collection and screening step will be performed by specially trained psychologists for the purpose of assessing whether participants meet the eligibility criteria for inclusion in the study. When a participant is found to meet the inclusion criteria, an independent researcher will implement randomization by telephone to avoid predictability Flowcharts giving an overview of the study design and the study timeline are summarized in Figs. 1 and 2, respectively.

Inclusion criteria will be as follows: (a) older than 18 years of age; (b) no psychiatric diagnosis (self-reported); (c) more than 1 year’s experience of daily meditation practice, (d) having a computer and Internet connection at home; (e) being able to read and understand the Spanish language; and (f) willingness to participate in the study and sign the written informed consent form. Exclusion criteria include the following: (a) any diagnosis of a disease that may affect the central nervous system (pathological condition affecting the brain, traumatic brain injury, dementia) or other psychiatric diagnoses or acute psychiatric illnesses (severe range of depression, substance dependence or abuse, history of schizophrenia or other psychotic disorders, eating disorders), except for anxiety disorder; (b) any medical, infectious or degenerative disease that may affect mood; presence of delusional ideas; and hallucinations consistent or not with mood and suicide risk; and (c) taking any psychiatric medication.

### Sample size

The sample size estimation was based on testing whether the trend in the pre-to-post change would be different between the intervention groups. Firstly, we assumed that mindfulness practice would be able to present small pre-post effects on deconstruction of the ego, according to the previous studies on this subject [21]. For this reason, we assumed a common mean (SD) at baseline of 65 in the Nondual Embodiment Thematic Inventory [22], and a mean at post-test of 69 and 75 in the mindfulness group and the other deconstructive practices, respectively. This assumes a pre-post standardized difference in the mindfulness group of *d* = 0.3, a pre-post standardized difference in any of the other deconstructive practices of *d* = 0.8, and a post-test standardized difference between the mindfulness group and any of the other arms of *d* = 0.5, which corresponds to the average effect in some exploratory studies conducted by our group [23] and is aligned with the 0.5 standard deviation criterion considered clinically relevant [22]. Using a 1:1:1:1 ratio, assuming a 5% significance level and a statistical power of 80%, and applying the Hotelling-Lawley Trace approach for the general linear model, which supports the inclusion of baseline covariates and coincides with the Wald test for the general linear mixed model [24], we will need a total of 240 participants, and thus 60 subjects in each group.

### Randomization and blinding

Participants who meet the inclusion criteria for the study will receive a code from the screening assessors in order to preserve their confidentiality. An independent researcher unaware of the study characteristics will perform the randomization process. In order to randomly allocate the participants to one of the four previously described conditions, a computer-generated random number sequence (https://www.randomizer.org) will be used by means of a simple allocation strategy. After the participants are informed of the study characteristics, they will agree to participate before random allocation and without knowing the condition to which they will be allocated. The researcher who administers baseline assessments will be blind to the participants’ practice group. This researcher will be different from the one—also blinded—who administers the other measures throughout the study. Participants will complete all the measures online. Participants will not be blind to the intervention condition for practical reasons, but they will be blind at the time of the first assessment (pre-intervention). The person responsible for carrying out the data analysis will not know the type of intervention that each participant has carried out.

### Data management

A psychologist from the research team will manage and monitor the data. All study information will be kept in secure drawers with limited access. Electronic data files will be password protected. Participant codes and personal information will be stored in a separate password-protected file. The data will be stored for 5 years after the end of the study, and only the study investigators led by the principal investigator will have access to the final data set. Results of the study will be presented through peer-reviewed publications and conferences. In addition, participants who request it will receive a report with the final results.

### Interventions

Four different meditative interventions will be assessed: (a) mindful breathing, which will be considered the control condition; (b) prostrations, according to Tibetan Buddhist tradition, (c) The Koan Mu, according to Zen Buddhist tradition, and (d) the mirror exercise, according to Toltec tradition. All participants in the four arms will receive these general instructions:

- Formal practice should take 30–60 min/day. It can be divided into as many as 4 sessions/day at times of participants’ choosing, but the recommended times are after waking up in the morning and before going to bed at night.
- There is no limit to the number of times informal practice can be performed during the day.
- Use of a diary is necessary to record the time and duration of all formal and informal practices.
- The intervention will have a duration of 60 days. During this period, participants are to take part only in the intervention to which they have been randomized and no other. After this period of time and during the follow-up, participants will be able to practise any kind of meditation and at times of their choosing, but this information must always be recorded in their diary.

Specific instructions for every intervention:

1. Mindful breathing (I): paying attention to your breathing

- Step 1. Assume a comfortable sitting posture, allowing the body slowly settle into position. You can take one or two deep breaths to draw your attention to your body. Then very gradually begin to notice your bodily sensations at that time (for example, your body making contact with the floor, mat or chair; the temperature of your skin; general sensations), until you come to include all the sensations of your entire body in general.
- Step 2. Very slowly start to become aware of your breathing. You can focus your attention on the movements of your chest or abdomen as you breathe in and out, or on the sensations of the air entering and exiting your nostrils as you breathe. It is important to follow the natural flow of your breathing, without trying to change it, simply observing and being aware of it, accepting what is happening in the present.
- Step 3. Your mind will eventually begin to wander. It is quite normal for your attention to be repeatedly distracted from your breathing and bodily sensations. This is not a problem. When you become aware of a certain distraction arising in your mind—whether in the form of a sensation, thought, feeling or sudden impulse—simply be aware that the mind is wandering, and allow the distractions to pass kindly, without becoming upset with yourself or judging them, and then redirect your attention to your breathing and your whole body.
- Step 4. You may experience intense sensations, such as pain in a part of your body, and you will find that your attention is repeatedly drawn away by such sensations from your chosen anchor (breathing or body). You may want to take advantage of this moment to deliberately decide to change your posture or to keep it and change the focus of your attention to those more intense sensations. If you choose this option, you can explore the sensations in more detail: How do you feel those sensations? Where? Do they change intensity or location over time? The aim is not to think about these things but what you feel. You can use your breathing to direct your attention to the area, by “breathing it”, as you would when practising body scan meditation.
- Step 5. Whenever your attention is drawn away by the intensity of the physical sensations or when your attention is distracted, you can reconnect with the present by directing your awareness to the movement of your breathing or the sensations of your whole body. Your awareness can then be expanded to take in all the sensations coming from all parts of your body.
- Step 6. Before the session ends, turn your attention back to the sensations of abdominal breathing. You can feel the connection with your inhaling and exhaling, instant by instant, perceiving your how you are anchored by your breathing and the feeling of balance. Slowly, when you feel ready, you can bring the practice to an end.

Mindful breathing (II): paying attention to your body

(Between steps 5 and 6)

After 10–15 min of mindful breathing, as previously described, allow your awareness to expand to all the sensations of your whole body. Gradually change the focus of your attention by moving away from your breathing and becoming aware of the sensation of your whole body and of how your sensations are continuously changing. You could feel your breathing throughout your body, as if it were breathing.

Besides your whole body and the sensations of breathing, you can include the sensations of the parts of your body in contact with the floor, chair, clothes or any other object.

If your mind wanders, which is its usual behaviour, be happy that you have become aware of this, observe where your mind has gone, and draw it back kindly to the sensation of your whole body.

If you experience unpleasant or painful sensations, follow the instructions previously given in steps 4 and 5.

When you are ready, you can bring the session to a close as described in step 6.

Mindful breathing (iii): paying attention to sounds, thoughts and emotions

(Between steps 5 and 6)

• Sounds. Turn your attention to sounds. Get a feeling for where they come from, what their characteristics are, how they appear and disappear, where in the space around you the come from (above, below, besides, in front or behind you).

You need not follow the sounds; they happen without you having to seek them out. Do not think about them or label them; only experience them as simple auditory sensations (i.e. pay attention to their tone, pitch, volume and duration). Even if you hear words in your own language, do not think about their meaning; listen to them as if they were in a different language. Be aware of the more and less intense sounds, and of the space between sounds and of silence.

If your attention wanders, acknowledge kindly where it has gone and bring it back to the sounds. This will happen repeatedly.

Attention to sounds is a very powerful practice for expanding your awareness. You can continue with mindful breathing and move on to mindfulness of thoughts and sensations, or this can be a separate practice on its own.

• Thoughts and emotions. Just as you have focused your attention on sounds, which are phenomena beyond your control and appearing in your mind autonomously, the thoughts that occur during meditation (unlike when you think voluntarily) are also beyond your control; they simply appear. Observe the process of how they appear, how they remain in your mental space, and how they disappear in a few seconds, without you having to do anything. You can even perceive the side of your mind in which they appear: from above, below, on the right or on the left. Do not try to stop them from appearing, speed up the process or wish them to disappear. Let them follow their natural process, as you did with the sounds.

Some people find it useful to imagine their mental space as a cinema screen and observe how thoughts appear on it, using the same process previously described. Another popular metaphor is to imagine your mind as the endless blue sky and thoughts as clouds that appear in it.

- Attention without an object. To finish it, you might want to let go of the object on which you were focusing your attention (breathing, sounds, thoughts) and allow your field of awareness expand to include everything that appears in your mind and body. Simply try to relax your awareness, without anchoring it. Do not latch on to anything or want anything; simply observe what is happening with equanimity. When you are ready, you can bring the session to a close (as described in step 6).

As an informal practice, focus your attention on your breathing whenever you can during the day.

(2) Postrations

The term “prostration” is generally used to describe an act of homage and respect. In Tibetan Buddhism, which is the most widespread form of *Vajrayana* Buddhism, the tantric path begins with special, or extraordinary, preliminary actions. These special preliminary actions are divided into five parts. The first of these is the Refuge practice, which is typically accompanied by prostrations. As explained by Kalu Rinpoche in his book *Foundations of Tibetan Buddhism*, prostrations are the physical element of the physical, verbal and mental aspects involved in the Refuge [25]. This practice includes a visualization (mental activity), a recital (speech activity) and a gesture/movement (bodily activity).

According to Patrul Rinpoche, as explained in his best-known book *Words of My Perfect Teacher*, prostrations are the antidote to pride, and it is important to combine the body, words and mind when performing them [26]. Therefore, the goals of this practice are to dissolve pride, purify the physical, verbal and mental dimensions, and unify and integrate these three spheres of the person.

Performing prostrations:

The practice begins with visualization. In this context, we have reduced it to the essentials in order to facilitate the practice. Visualize Shakyamuni Buddha in front of you, feeling that he is truly present. Visualize all beings beside you, feeling that they are not essentially different from you and that they are prostrated together with you. You should visualize the images with the consistency of a rainbow or hologram, not as solid entities. This should not diminish the feeling of their presence.

In the physical aspect, you start by cupping your hands to form the shape of a closed lotus flower that is about to open, leaving an empty space between them. The palms should not be pressed together without leaving a space, nor should they only be joined at the fingertips.^2^ Place your cupped hands on your head first, then at your throat and, finally, at your heart to purify the corruptions of the body (e.g. the actions of killing, stealing or harmful sexual conduct), the words (e.g. lying, causing arguments, harmful words or deceitfulness) and the mind (e.g. envy or covetousness, wishing harm on others). Next, touch the floor with five points of the body: the forehead, both palms of the hands and both knees.^2^ Once you have finished stretching out your body fully on the floor, you can raise your cupped hands over your head. Afterwards, when you stand up again, you straighten your body completely, cupping your hands again. Continue to perform prostrations in this way.

With regard to the words, each lineage recites their own verses, which in this case have been adapted to a simplified formula: “All beings and I prostrate ourselves before Buddha, who has transcended suffering. May all beings be able to transcend suffering.” This is recited while performing each prostration.

As the practice progresses, you can visualize how rays of light emanate from Buddha to tough your forehead, throat and heart, purifying your body, words and mind.

Quality is more important than quantity, the main indicators of which are the level of integration between the physical, verbal and mental actions and the feeling of presence in the visualization.

As informal practice, you can repeat the verse in your mind and perform the visualization whenever you can.

(3) Koan Mu

*Koan* is a Japanese word for dialogue or question. It can be considered the hallmark practice of the schools of Zen Buddhism, mainly of Rinzai Zen. In this meditation, the student observes or concentrates on a single word or phrase for days or weeks. There are several books with collections of koans in the Zen tradition, and a type of progression has been described and used by meditation masters. One of the most famous ones that has been used by hundreds of masters for centuries with beginners is “Mu” or “Does a dog have Buddha-nature?”.

The koan is explained to the students in this way (Japanese version):

A monk asked Joshu: “Does a dog have Buddha-nature or not?” Joshu replied, “Mu” (Mu is a Japanese word that can be translated as “No”).

The practice as proposed to the participants will be based on the instructions given by Zen Master David Loy at a retreat, as follows:

Assume your usual meditation posture. After taking several conscious breaths, gradually make contact with “Mu”, while maintaining unforced breathing. When you breathe in, only observe the mind, without generating anything within. When you breathe out, mentally pronounce “Muuuu” with a deep and low-pitched sound, in tune to the rhythm of your breathing. Forget all notions of obtaining an answer, much less something a rational one. And breathe out again producing mentally the sound “Muuuu”. Do not look for anything; eliminate any hopes or expectations; there is nothing to gain or win. Do not expect anything in the future; focus on what you are doing just now. Take this to your everyday life, not only during formal meditation but in informal practice as well: while you are walking, eating, showering or in any other activity.

(4) The mirror exercise

According to the Toltec tradition, when we dream, we are the same figure that we are in our waking reality. This single figure makes us repeat ourselves both when we are awake and in our dreams. One of the first tasks is to break the relationship between our face, i.e. our ego, and our past. The way in which we do this is to wear a mask in front of a mirror. In the Toltec tradition, this practice is called “the venerable quetzal”. The quetzal is one of the sacred birds of Mexico. When it flies, it loses its green colour and takes on iridescent colours. The practice aims to make us lose our ego and our past in order to become beings of light. Two different masks are used. The mask can be changed two or three times each session, so you we will need at least two masks. We use our intuition to make the change.

The practice consists of sitting in front of a mirror, putting on a mask and telling the story of your life while wearing the mask. You have to explain your problems and concerns aloud, as if we were telling it to a psychotherapist. You have to speak about your problems and worries until you feel free from your present life. You should dedicate between 30 and 45 min to the exercise every day, whenever you can.

The result of the practice tends to be that by the 15th day, you discover that you no longer believe the story of your life and that it does not even matter to you. By changing masks, you learn to laugh at your own life. It reaches the point where you have nothing more to say, and you stand in front of the mirror changing masks. Nothing in your life makes you feel bad. Dreams tend to change to greater lucidity.

As informal practice, you should observe yourself in front of a mirror whenever you can. Masks are not required for the informal practice.

### Measurements

Evaluations will be performed at screening, pre-intervention, post-intervention and 3 and 6-month follow-up. All variables and assessment periods of the study can be found in Table 1.

**Table 1:** Study outcomes

Instrument	Assessment area	Time to assessment
Sociodemographic	Gender, age, marital status, education, occupation, economic level	Screening
NETI	Deconstruction of self	Baseline, post-treatment and follow-up sessions
NADA	Nondual Awareness	Baseline, post-treatment and follow-up sessions
FFMQ	Facets and factors of mindfulness	Baseline, post-treatment and follow-up sessions
PHI	Well-being	Baseline, post-treatment and follow-up sessions
SOCS-O and SOCS-S	Compassion	Baseline, post-treatment and follow-up sessions
PANAS	Positive and negative affect	Baseline, post-treatment and follow-up sessions
OAV	Altered states of consciousness	Baseline, post-treatment and follow-up sessions

*NETI* Nondual Embodiment Thematic Inventory, *NADA* The Nondual Awareness Dimensional Assessment, *FFMQ* Five Facet Mindfulness Questionnaire, *PHI* Pemberton Happiness Index, *SOCS-0 and SOCS-S* Sussex-Oxford Compassion Scales, *PANAS* Positive and Negative Affect Schedule, *OAV* Altered State of Consciousness Rating Scale

#### Sociodemographic data and meditation experience

Participants will be asked about their gender, age (in years), nationality, current city of residence, marital status (single, married, cohabiting couple, separated/divorced, widowed or other), education (undergraduate/bachelor’s degree/diploma, master’s degree, doctorate and others) and for information regarding their experience with meditation, such as whether they have had meditation experience in general (“yes” or “no”), the mean in minutes for each time that they practised formal meditation, length of meditation experience (i.e. less than 1 year, between 1 and 3 years, between 4 and 6 years, between 7 and 9 years, or more than 10 years), the time (in months) that they may have interrupted their formal practice during their lives, and the percentage of their time dedicated to each type of formal meditation (concentrative or focused attention, open monitoring, compassion/loving-kindness, values, deconstructive/non-duality and informal practice). A brief explanation of each type of meditation practice will be provided. In addition, an open field is included so that participants can indicate other meditative techniques that they practice and have not been explicitly collected. The meditation information provided and collected will be based on author recommendations and previous studies [11, 27].

#### Main outcome

The Nondual Embodiment Thematic Inventory (NETI will be the primary outcome at post-intervention). This is a 20-item questionnaire designed to evaluate qualities of non-dual experience and spiritual awakening. The NETI assesses a number of facets, including frequency of non-dual experiences, which refer to self-transcendent states that have also been referred to as peak experiences (e.g. “Understanding that there is ultimately no separation between what I call my “self” and the whole of existence”) [28]. The items are rated on a 5-point scale with a score of 1 indicating “never” and 5 indicating “All of the time”. A total score, ranging from 20 to 100, is calculated by totalling the scores from all the items, with higher scores indicating higher levels of non-dual awareness. The scale has shown good psychometric properties in its original validation, with an internal consistency of Cronbach’s alpha = 0.91 [28, 29].

#### Secondary outcomes

##### The Nondual Awareness Dimensional Assessment (NADA)

This scale was developed to measure experiential effects of nondual awareness, such as bliss and self-transcendence. To study the trait, there is the NADA-T version with 13 items (e.g. “I have experienced all notion of self and identity dissolve away”), which are rated on a 5-point Likert-type scale ranging from “never or very rarely” to “very often or always”. On the other hand, to assess the state, the NADA-S version composed of 3 items and scored on a 10-point Likert scale (1 = Not at all, 10 = Very Much) will be used. Their psychometric properties are adequate (NADA-T: Cronbach’s alpha > 0.9; NADA-S: Cronbach’s alpha between 0.67 and 0.88) [30].

##### The Five Facet Mindfulness Questionnaire (FFMQ)

With 39 items (range 39–195 points) and five subscales: observing (e.g. “I am aware of the bodily sensations when O take a bath”), describing (e.g. “I find it hard to express what I feel”), acting with awareness (e.g. “I am easily distracted”), non-judging of inner experience (e.g. “I am aware that some of my thoughts are not normal, and I know that I shouldn’t feel that way”) and non-reactivity to inner experience (e.g. I think before reacting under stressful situations”). The items are rated on a Likert-type scale ranging between 1 and 5 points (from 1 = “never or very rarely true” to 5 = very often or always true”), and higher total values indicate better full mindfulness. The validated Spanish version of the questionnaire that has demonstrated appropriated psychometric properties will be used (Cronbach’s alpha > 0.80) [31, 32].

##### Sussex-Oxford Compassion Scales (SOCS)

The Sussex-Oxford Compassion for Others Scale (SOCS-O) and Sussex-Oxford Compassion for the Self Scale (SOCS-S) are two 20-item self-report scales measuring compassion. These are based on the theoretically and empirically supported definition of compassion as comprising five dimensions: (a) recognizing suffering (e.g., “I recognize when other people are feeling distressed without them having to tell me”), (b) understanding the universality of suffering (e.g. “I understand that everyone experience suffering at some point in their lives”), (c) feeling for the person suffering (e.g. When someone is going through a difficult time, I feel kindly towards them”), (d) tolerating uncomfortable feelings (e.g. “When someone else is upset, I try to stay open to their feelings rather than avoid them”) and (e) motivation to act/acting to alleviate suffering (e.g. When others are struggling, I try to do things that would be helpful”). The items are rated on a Likert-type scale ranging between 1 (“not at all true”) and 5 (“always true”) points. Their psychometric properties are adequate (SOCS-O: Cronbach’s alpha between 0.76 and 0.97; SOCS-S: Cronbach’s alpha between 0.74 and 0.97) [33].

##### The Pemberton Happiness Index (PHI)

The PHI is used to assess remembered well-being (general, hedonic, eudaimonic and social well-being) through 11 items rated on a Likert scale ranging from 0 (strongly disagree) to 10 (strongly agree). In addition, experienced well-being (positive and negative emotional events that may have happened the day before) is also measured through 10 items answered dichotomously (“yes” or “no”). The remembered well-being score is calculated with the mean score of the 11 items and varies from 0 to 10. The items for experienced well-being are transformed into a single score from 0 (zero positive experiences and 5 negative experiences) to 10 (5 positive experiences and no negative experiences). To calculate the overall PHI index, which included remembered and experienced well-being, individuals’ scores of the 11 items related to remembered well-being plus the sum of scores on the experienced well-being were summed; the total sum is then divided by 12, so the resulting PHI total mean score also ranges from 0 to 10. The Spanish validated version will be used in this study (Cronbach’s alpha > 0.80) [34].

##### Positive and Negative Affect Schedule (PANAS) [35]

This questionnaire comprises 20 items and two independent dimensions: positive affect (e.g. “interested”) and negative effect (e.g. “irritable”), with answers ranging in a Liker-type scale from 1 “very slightly or not at all” to 5 “extremely or very much”. Each scale has 10 items, and the score range for each is from 10 to 50. The Spanish version of the PANAS, adapted with adequate psychometrics and designed to assess affective states over the last week, will be used (Cronbach’s alpha between 0.87 and 0.91) [36].

##### Altered state of consciousness rating scale (OAV)

This is a 42-item scale grouped into 11 domains (i.e. experience of unity, spiritual experience, blissful state, insightfulness, disembodiment, impaired control and cognition, anxiety, complex imagery, elementary imagery, audio-visual synaesthesia and changed meaning of perceptions). Participants are asked to respond to the described experiences by placing marks on horizontal visual analogue scales (VAS), which are anchored as *no, not more than usual* on the left and as *yes, very much more than usual* on the right. It assesses altered states of consciousness produced by drugs such as ketamine, psilocybin or MDMA and the effects of meditation. Their psychometric properties are adequate (Cronbach’s alpha > 0.80) [30, 37].

##### Diary for recording activity

During the intervention and follow-up periods, participants will be asked to record information on the formal practice (number of sessions, total duration, time of day) and on informal practice (duration, time of day) for inclusion as a moderator variable.

##### Interconnection graph

After each meditation, the participants will record the degree of connection they feel with the “community”, “humanity” and the “planet/world” using a pictorial scale. Each item will be evaluated from seven pairs of superimposed circles representing different levels of interconnection, with one circle representing the participant and the other circle representing one of the three items mentioned. The participant must highlight which pair of circles, respectively, represents their feeling of connection with each item.

### Statistical analysis

Descriptive data will be visually compared in terms of sociodemographic data, meditation experience and psychological variables across the arms at baseline to ensure balance among groups. They will be summarized using means (SDs) for continuous variables, and frequencies (percentages) for categorical variables. All analyses will follow a pre-specified plan. Outcomes will be compared between groups using the intention-to-treat principle. Missing outcome data (assumed to be missing at random) will be imputed using multiple imputations based on chained equations. The imputation model will include the outcomes at each time point, trial arm, the completion of the training and the extent to which meditation was practised, as well as other characteristics pre-specified for covariate adjustment, generating 20 imputed datasets. The primary analysis will be carried out for NETI total scores at post intervention. Outcomes at each time point will be compared using mixed-effects linear regression models. The main comparisons will be adjusted for baseline scores, gender and age. Means (SDs) will be reported for each trial arm along with the adjusted mean difference between arms, 95% confidence interval (95% CI) and *p*-value for the adjusted mean difference. Results from the complete-case analyses will be also reported, as well as additional sensitivity analyses utilizing complier-average causal effect (CACE)/instrumental variable methods to explore the causal effects of (a) participant engagement with the intervention and (b) regular meditation practice.

An alpha level of 0.05 will be established using a two-tailed test. The probability values relative to the main analysis will be adjusted according to Benjamini-Hochberg’s correction for multiple comparisons, but the secondary analyses will not be corrected.

### Qualitative analysis

A qualitative study is proposed in the context of this RCT. Through in-depth interviews [38], an attempt will first be made to understand the experiences of people practising types of deconstructive meditation, the expectations and difficulties with these practices, and the reasons why people decided to end their practice. Focus groups [38] will also be formed to explore dynamic interactions in light of cultural characteristics and specific values that can be the basis for participants’ views and preferences. In this way, qualitative information will be obtained from a collective and group perspective that only emerges from individual interviews with some difficulty [39]. This methodological triangulation will increase the consistency and rigour of the study by combining multiple techniques [40].

#### Participants

Participants in the qualitative study will be recruited from the participants of the RCT. They will be selected while considering the variables that maintain homogeneity and discursive heterogeneity regarding the aims of the study, such as age, gender, previous meditative experiences, time devoted to meditation or changes in the measures assessed. Through purposive sampling, an attempt will be made to capture rich and varied information in agreement with the goals of the study [41], thus optimizing the representation of participants’ opinions.

A preliminary analysis will allow us to progress though the interviews in an iterative manner until data saturation; in other words, until the new information becomes redundant and provides no new perspectives. We expect that this will occur after approximately 10 interviews in each of the four groups of meditation assessed. After the interviews, four focus groups will be conducted, each consisting of between eight and twelve subjects. An attempt will be made to ensure adequate representation of the stratification variables considered but with intergroup heterogeneity and intragroup homogeneity to allow the groups to be formed appropriately [39, 42]. The information to establish stratifications will be collected before beginning the individual/group interviews.

#### Data collection

Topics to be addressed in both the individual and group interviews will be discussed by the team of researchers and will guide the interviewers/moderators in the same direction. In-depth interviews will be carried out by a single interviewer, who will indirectly raise the objectives of the study, questioning interviewees about topics in an open and progressive way. The interviewer will also explain the need to record the session (only audio) and will take notes about non-verbal language elements. The focus groups will be moderated by an interviewer, and another person from the research team may be present as an observer. The role of the moderator will be to indirectly explain the aims of the study, introduce the topics of interest, and direct group dynamics in order to encourage dialogue and participation. The function of the observer will be limited to collecting field notes to provide additional information to the verbal data, such as information related to non-verbal language, responses to the moderator’s interventions and contextual aspects. The indirect introduction of the objectives will enable us to analyse the participants’ natural approach to the issues of interest.

We will seek to achieve optimum standardization in the sessions with a specification guide whose content will be open and flexible (the topic list is included in Table 2), while allowing the inclusion of emergent issues introduced by the participants [43].

**Table 2:** Topic list

Areas	Issues
Characteristics of the practice	Culture shock
	Routine formal and informal practice
	Enjoyment/boredom
Experiences	Peak experiences
	Lucid dreams
	Intrasession
Expectations	Pre-practice
	Post-practice
Information	Need for a facilitator
	More/less information
Results	Description
Altered states of consciousness	Description

We will ensure the confidentiality and anonymous nature of the study for potential participants. All the sessions will be audio taped with the consent of the participating subjects, and materials will be verbatim transcribed. Both interviews and focus groups will last around 60–90 min. The two researchers responsible for the focus groups will meet after each session to clarify and exchange views and field notes. This information will be analysed with data from the audio recordings and notes [43].

#### Analysis strategy

The body of text will consist of the verbatim transcriptions of the recordings, supplemented by notes from observations and comments by interviewers/moderators. The interviewers/moderators will verify the transcripts to guarantee the accuracy of the data. Final validation will be performed by inviting the participants to read and discuss the transcribed content [44].

The preliminary analysis will begin after the first interviews and progress through the interviews iteratively to confirm or discount the topics found [45]. The transcriptions and notes will be reread and analysed by two independent social researchers who are experts in qualitative content analysis, using a vertical, interpretive, emergent and non-frequency-based approach [46]. Through thematic analysis, supported by the initial topic guide for content specification as the first framework for approximation and using the constant comparative method from grounded theory, key units of meaning will be identified that enable us to deconstruct every sentence [47]. The information contained in the generated text segments will be compared and grouped through open coding until a common conceptual denomination for all segments of text that share the same thematic unit is reached [48]. Provisional interpretations will be made to highlight the characteristics and relationships of the emerging codes generated, which will form new categories as a result of their gradual fusion [47–49]. These broader categories, defined in an exclusive manner based on agreement among the researchers, will make the conceptual structure denser until a parsimonious solution can be reached. Cases that cannot be classified will be actively sought out during the analysis, and the emerging categories will be redefined in response to these cases [50, 51].

The information gathered from focus groups will be analysed using the model of sociological analysis [52]. In this technique, the reading and organization of data are not based on fragmentation of the text but on the interpretation of the different discursive positions among the participants and the identification of the explanatory axes of their interventions. Themes and patterns will be identified and coded by two independent researchers, considering the context in which the interventions took place. Each step towards configuring the potential explanatory axes will require an iterative reading to validate and confirm the interpretation of new findings, ensuring the credibility of the process by reviewing the data again from the beginning [53]. At first, the material from different groups will be analysed separately (intragroup analysis). However, it will subsequently be regrouped and pooled in order to compare the relevant themes (intergroup analysis). The precision of the analysis will be increased by highlighting the consistent results in all groups, and special attention will be given to “sensitive moments” in the interaction as indicators of important topics. The whole process will be triangulated between the two researchers, and differences will be resolved through discussion.

To manage the data, we will use the MAXQDA 2022 data analysis software package. The results of the thematic content analysis of the interviews and the interpretation of the main explanatory axes in the focus groups will be presented alongside the empirical references in the text, selecting the most representative verbatim segments for use as examples. The researchers will develop a list of concepts identified in the interviews and focus groups and create conceptual diagrams that increase understanding and comparability. The final coding framework will be discussed with the main researcher and two participants from individual/group interviews, so that all stakeholders agree.

### Patient and public involvement

Although importance is being given to engaging stakeholders in the choice of research questions, no members of the public were directly involved in the development of the research questions, in this case because of the novelty and incipient use of deconstructive meditative practices on improving well-being in the general population. Nevertheless, a group of participants will be contacted after the trial and will be interviewed about the research questions as well as the design and feasibility of interventions. This qualitative information will be used as an advisory first experience of public involvement and its responsiveness in order to develop and refine future research related to the use of deconstructive meditative practices in the general and even clinical populations.

## Discussion

This exploratory randomized controlled trial will allow us to know more about the efficacy of several deconstructive meditation techniques in order to improve the well-being of healthy people. Possible mechanisms of action and the relationship with time of formal and informal practice may also be studied. Finally, the association of any of the practices with altered states of consciousness will be made clearer. The qualitative study will provide information on the barriers and facilitators for Western meditators to adapt ancient meditative practices that are deeply entwined in Eastern culture and religion. The field of psychology could benefit from specific interventions developed over generations to increase the mental health, wellness and happiness of humanity, and to generate prosocial and interconnected attitudes and behaviours in a frantic world.

The study has various strengths. Firstly, to the best of our knowledge, this is the first RCT to assess different types of deconstructive meditation applied to improving well-being, and the first qualitative study on the expectations of, effects on and barriers to participants. Secondly, in spite of the exploratory character of the study, the sample size is large enough to generate knowledge and hypotheses on the comparative efficacy of all the types and on the differences in their mechanisms of action.

The limitations are the potential non-representativeness of the sample, the multiple comparisons between arms that would limit statistical power, and the relatively short follow-up, all of which are aspects related to the exploratory nature of the study.

## Ethical aspects and dissemination

This study will be conducted according to the international standards of the Declaration of Helsinki and subsequent amendments. This trial will be conducted in compliance with the study protocol and with good clinical practice guidelines with the main aim of protecting and preserving human rights [54]. Data security will be guaranteed; the participants included in the study will be protected by the Organic Law on Protection of Personal Data (15/1999 of December 13, LOPD) and all relevant EU legislation in this regard; and international privacy agreements will be observed and respected. The Internet platform will be accessed through a unique username + password combination. Since the interventions to be applied in this study are low risk, the creation of a Data Monitoring Committee is not considered necessary. This study has been approved by the Research Ethics Committee of the regional authority (CEICA Aragon, Spain registry number PI22/110). An informed consent form will be signed by participants before randomization. On the consent form, participants will be asked if they agree to use of their data should they choose to withdraw from the trial. Participants will also be asked for permission for the research team to share relevant data with people from the Universities taking part in the research or from regulatory authorities, where relevant. This trial does not involve collecting biological specimens for storage. The protocol will be available after its publication. Anonymised participant-level datasets and procedures will be available for replication studies on reasonable request.

## Clinical implications

Positive results of this RCT may have an important impact on the development of new interventions, not only for the improvement of happiness and well-being in healthy populations but potentially also for the prevention and treatment of psychological and medical disorders. The data on mechanisms of action could allow new interventions to be designed based on this knowledge, and the results of the qualitative study could help to tailor interventions to the characteristics of the individual. All this could increase the range and characteristics of the psychological interventions available for therapists, creating a new paradigm within third-generation psychotherapies.

## Trial status

The protocol version number is 2.0 which was approved on 23 March 2022. Recruitment will commence in September 2022 and is expected to be completed by September 2023.

## Data Availability

In accordance with the International Committee of Medical Journal Editors (ICMJE), the data generated by this trial will be made available upon reasonable request to researchers (i) who provide a methodologically sound proposal and (ii) whose proposed use of the data has been approved by an independent ethical review committee. The data sharing plan includes all of the individual anonymized and completely de-identified participant data collected during the trial, as well as other related documents such as the study protocol, the statistical analysis plan and the data dictionary with descriptive labels. Data will become available immediately following each publication with no end date and for any analytical purpose that is related to achieve aims in the original approved proposal. The database will be encrypted and password protected. Passwords will be provided by the corresponding author to interested researchers that meet the both previously described criteria. The plan and all related documents will be downloadable at https://doi.org/10.3886/E170741V1.

## Abbreviations

AUC: Area-under-the-curve
BDI-II: Beck Depression Inventory II
CBT: Cognitive behavioural therapy
CI: Confidence interval
CSRI: Client Service Receipt Inventory
EQ-5D: EuroQoL-5D
ETS: Expectation for Treatment Scale
FFMQ: Five Facet Mindfulness Questionnaire
GP: General practitioner
ICERs: Cost-effectiveness ratios
ICURs: Cost-utility ratios
ITT: Intention to treat
MBCT: Mindfulness-based cognitive therapy
MINI: Mini International Neuropsychiatric Interview V-5.0.0
OTS: Opinion of Treatment Scale
PANAS: Positive and Negative Affect Schedule
PC: Primary care
PHI: Pemberton Happiness Index
PHQ: Patient Health Questionnaire
QALY: Quality-adjusted life-year
REML: Restricted maximum likelihood
RM: Repeated measures
SF-12: Short Form Health Survey-12
TAU: Treatment as usual
WHO: World Health Organization
